# High prevalence of hepatitis A in indigenous population in north Brazil

**DOI:** 10.1186/s13104-020-05303-y

**Published:** 2020-09-29

**Authors:** Vanessa Salete de Paula, Flavio Augusto Pádua Milagres, Guilherme de Macêdo Oliveira, Juliana Custódio Miguel, Helena Medina Cruz, Leticia de Paula Scalioni, Vanessa Alves Marques, Monica de Avelar Figueiredo Mafra Magalhães, Anselmo Rocha Romão, Renata Gracie, Livia Melo Villar

**Affiliations:** 1 Molecular Virology Laboratory, Oswaldo Cruz Institute, FIOCRUZ, Rio de Janeiro, Brazil; 2 Federal University of Tocantins, UFT, Tocantins, Brazil; 3 Cancer Nacional Institute, INCA, Rio de Janeiro, Brazil; 4 Laboratory of Viral Hepatitis, Oswaldo Cruz Institute, FIOCRUZ, Helio and Peggy Pereira Pavillion, Ground Floor, Room B09, FIOCRUZ Av. Brasil, 4365 - Manguinhos, Rio de Janeiro, 210360-040 Brazil; 5 Hepatology Research Group, University of Plymouth, Plymouth, Devon UK; 6 Laboratory of Information in Health, Institute of Communication and Technological and Scientific Information in Health (ICICT), FIOCRUZ, Rio de Janeiro, Brazil

**Keywords:** Hepatitis A, Prevalence, Indigenous population, Vaccination

## Abstract

**Objectives:**

Little is known about hepatitis A virus (HAV) prevalence in indigenous communities. This study aims to evaluate the prevalence of HAV in indigenous community compared to urban population located at Western Amazon in Brazil.

**Results:**

A total of 872 serum samples were obtained from 491 indigenous and 381 non indigenous individuals aging 0 to 90 years. Samples were tested for total and IgM anti-HAV and positive IgM samples were tested for HAV RNA. The overall prevalence of total anti-HAV was 87%, increased according age showing 100% of prevalence in those aging more than 30 years (p < 0.0001) and it was similar among indigenous and urban population. Total anti-HAV prevalence varied between tribes (p < 0.0001) and urban sites (p = 0.0014) and spatial distribution showed high prevalence in homes that received up to 100 dollars. IgM anti-HAV prevalence was 1.7% with predominance in males, those aging more than 41 years. No HAV RNA was detected. In conclusion, high overall anti-HAV prevalence was found in indigenous communities in North Brazil demonstrating the importance of universal vaccination in this group.

## Introduction

In indigenous communities, the risk of infection by hepatitis A virus (HAV) is great, due to the poor sanitation conditions, non-potable water and frequent person-to-person contact that favor the occurrence of outbreaks [1]. Few studies have reported the prevalence of HAV in the indigenous community and values above 96% was observed in indigenous population in Brazil and Ecuador [1, 2].

Apinajé is an indigenous population located in the Western Brazilian Amazon since the eighteenth century in northern region of Tocantins and Araguaia rivers. This population include about 1409 indigenous people divided in 15 villages in 1500 km^2^ [3, 4]. There is a lack of data about HAV epidemiology in these communities that continue to have higher morbidity and mortality from many infectious diseases compared with the general populations in their countries [2, 5, 6].

This study aims to evaluate the prevalence of hepatitis A infection among an indigenous population of Apinajé ethnic located in Eastern Brazilian Amazon compared to urban population.

## Main text

### Methods

A cross-sectional study was conducted using a non-probability sampling method among Amerindians and non-Amerindians living in Eastern Amazon. Blood samples were collected after the approval of the Research Ethics Committee and the National Research Ethics Commission (CAAE: 32789914.6.0000.5248). In the village there was a responsible person (nurse or social worker) for providing data relating to individuals, such as date of birth and registration name. Blood samples were collected after clarifying the purpose of the study to each individual or guardian and the signature (written or digital) in the informed consent form. Eligibility criteria for participation of this study were: residence in the area and the provision of informed consent. Exclusion criteria were: confusion at the time of recruitment and disagreement with the terms of the informed consent.

According census of IBGE [7], there are 1768 indigenous in Tocantinópolis county. There is poor access to water and sanitation. Indigenous population are composed by the following communities: Girassol, Mariazinha, Riachinho, Prata, Serrinha, Folha Grossa. Non indigenous population are composed by Cacau, Mumbuco, Folha Grossa, Fazenda Bela Vista, Urban area Block 18, Urban area Block 22, Tocantinopolis Downtown.

Sample calculation was made using prevalence of total anti-HAV of 80% and degree of confidence of 95% with α of 0.05 and critical value of Zα/2 equal to 1.96. Considering, a population of 2591 individuals in the county, the minimal sample should be 225 individuals.

Blood samples were collected by venipuncture (10 mL) in tube without anticoagulants and stored on ice to transport to the laboratory. In the laboratory, the blood was centrifuged at 3500 rpm at 25 °C for 5 min. The supernatant was placed in previously identified microtubes with the registration number and stored at  20 °C, until processed at Viral Hepatitis Laboratory—Fiocruz/RJ.

Total and IgM anti-HAV antibodies were detected using commercial enzyme-linked immunosorbent assay according to the manufacturer's instructions using (ETI-AB-HAVK Diasorin® PLUS). Samples found to be negative on preliminary screening were considered seronegative. Samples that initially tested borderline or positive were retested using ELISA to confirm the results.

Positive IgM anti-HAV samples were submitted to TaqMan RT-PCR assay to quantify HAV genome as previously described [8–10]. RNA was extracted using Qiamp RNA viral mini kit (Qiagen). Reverse transcription and real time reaction were made using master mix containing 1× SuperScript™ III RT/Taq master mix (Invitrogen, Hammonton, NJ) and 1.25 µL of the assay mixture (300 nM of each primer, 150 nM probe) (Thermo, Assay, Foster City, CA). The synthetic curve, primers and probe were described previously [8, 10].

The socioeconomic variables for calculating the indicators proportion of monthly income per household were taken from the demographic census were taken from the population census 2010 [7].

Each individual gave information regarding age, gender and residential location and these data were included along to serological results into (Microsoft Excel) data spreadsheets. Prevalence was calculated for HAV and descriptive statistics were generated for the data.

The spatial representation of anti-HAV total and socioeconomic variables were based on cartography of the digital mesh of the 2010 census [7] and data of anti-HAV total according localities of the municipality of Tocantinópolis in the state of Tocantins. For the adaptation of the analyzed locations to the census tracts, the National Register of Addresses for Statistical Purposes of the IBGE was used [7]. Thematic maps of HAV was created using the Geographic Information System (GIS) ArcGis version10.4.

### Results

In the present study, 872 individuals were included aging 0 to 90 years, 491 were Indigenous and 381 were non-indigenous. Most of them were females (53.3%) and mean age of individuals was 27.4 ± 20.6 years. The socio-demographic characteristics of the 872 individuals included in this study are shown in Table 1.

**Table 1:** Hepatitis A virus markers among individuals from Amerindians tribes and urban areas of Tocantinopolis city

	Number tested (n = 872)	anti-HAV + (n = 757)	HAV IgM + (n = 15)
Total population studied	872 (100%)	757 (87.0%)	15 (1.7%)
Sex			
Female	467 (53.5%)	415 (88.8%)	05 (1.1%)
Male	405 (46.5%)	342 (84.4%)	10 (2.4%)
Age group (years)			
0 a 2	52	18 (34.6%)	0 (0.0%)
3 a 5	72	33 (45.8%)	0 (0.0%)
6 a 12	137	110 (80.2%)	0 (0.0%)
13 a 21	170	159 (93.5%)	2 (1.2%)
22 a 30	132	128 (96.9%)	1 (0.75%)
31 a 40	99	99 (100.0%)	2 (2.0%)
41 a 50	69	69 (100.0%)	3 (4.3%)
51 a 60	58	58 (100.0%)	1 (1.7%)
> de 61	83	83(100.0%)	6 (7.2%)
Location			
Indigenous			
Prata village	51	49 (96.0%)	0 (0.0%)
Girassol village	66	63 (95.5%)	0 (0.0%)
Mariazinha village	155	115 (74.2%)	0 (0.0%)
Riachinho village	18	16 (88.8%)	0 (0.0%)
Serrinha village	64	60 (93.7%)	0 (0.0%)
Folha Grossa village	137	130 (94.9%)	13 (9.5%)
Non-indigenous (urban areas)			
Cacau urban area	62	48 (77.4%)	1 (1.6%)
Mumbuco urban area	83	70 (84.3%)	0 (0.0%)
Urban area block 18	84	76 (90.5%)	1 (1.2%)
Urban area block 22	62	47 (75.8%)	0 (0.0%)
Bela Vista urban area	12	8 (66.6%)	0 (0.0%)
Tocantinopolis downtown	78	75 (96.1%)	0 (0.0%)

Total anti-HAV prevalence was 87% (757/872) where prevalence up to 50% was observed in individuals aging below 5 years and 100% of prevalence was found in those aging more than 30 years showing statistical significance (p < 0.0001). Total anti-HAV was detected in 433 Indigenous (88.2%) and 324 urban population (85.0%) and it was not statistically significant (p = 0.189). Gender was not associated to anti-HAV prevalence (p = 0.05). Total anti-HAV prevalence varied between tribes (p < 0.0001) and between urban sites (p = 0.0014). In indigenous population, the lowest prevalence was found in Mariazinha village and among non-indigenous population, the lowest prevalence was found in Bela Vista Urban area.

**Fig. 1:**
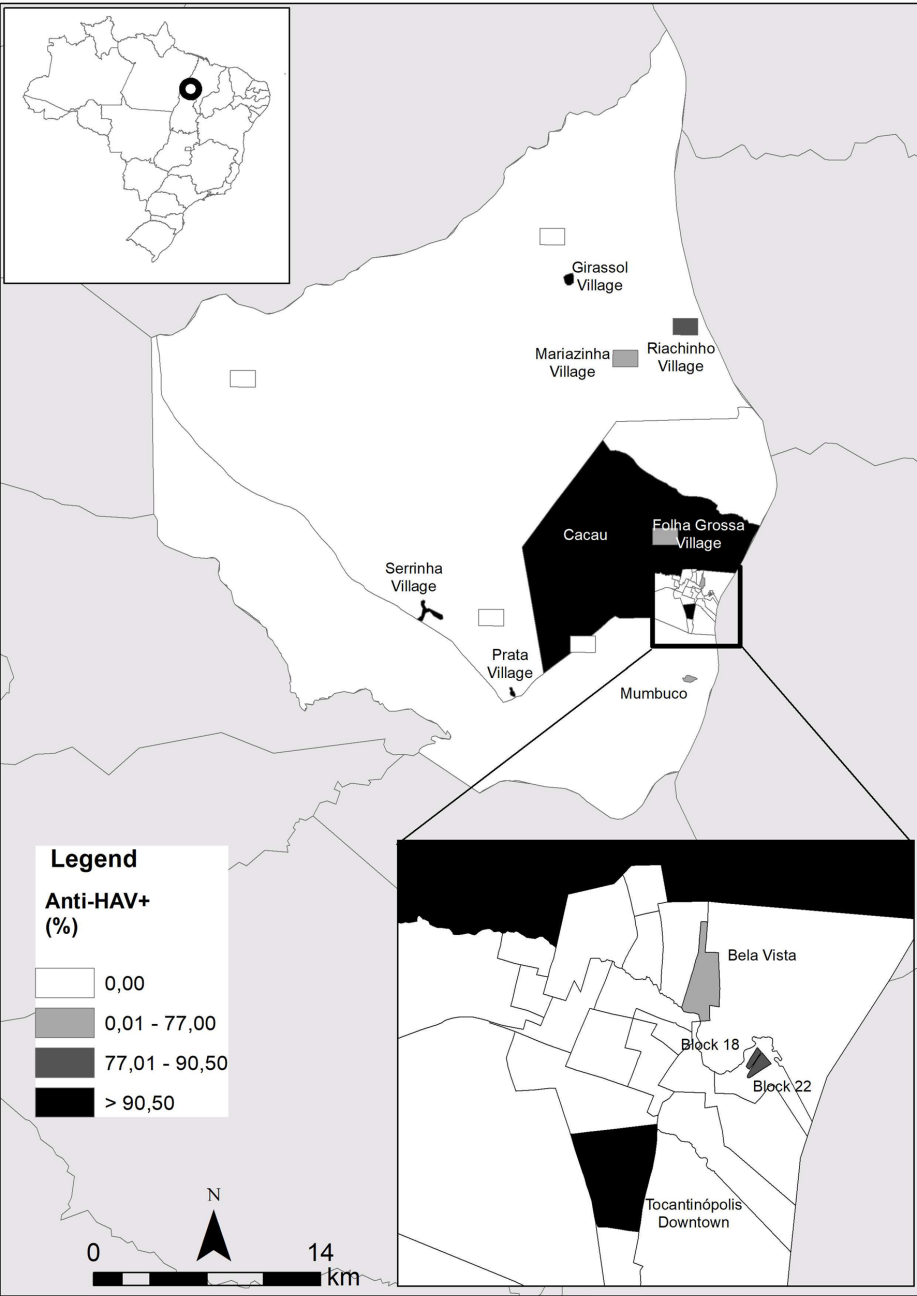
Prevalence of cases of previous HAV infection (total anti-HAV) according site of recruitment in Eastern Amazon

**Fig. 2:**
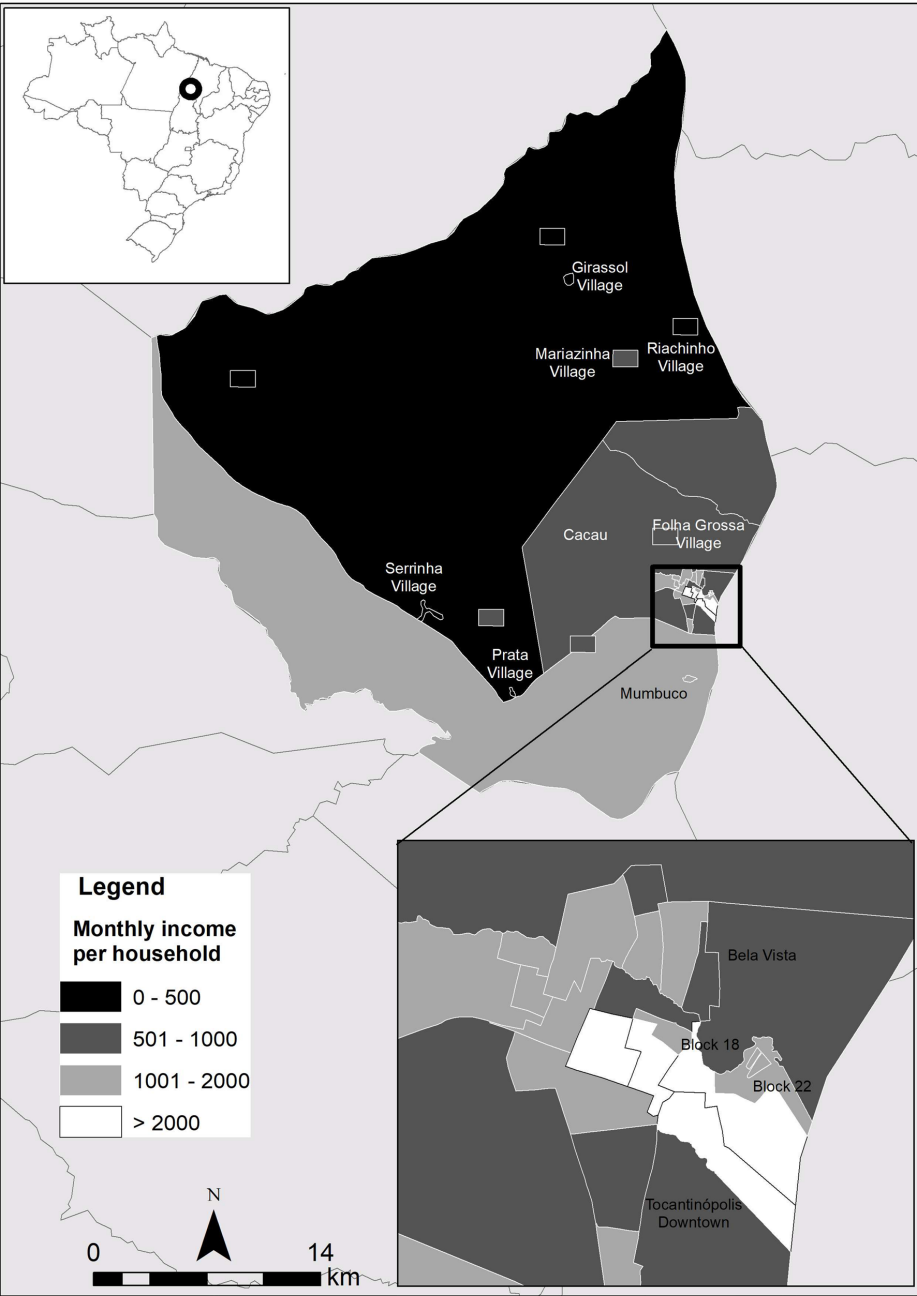
Distribution of monthly income per household according site of recruitment in Eastern Amazon

In Fig. 1, it was possible to observe that the highest total anti-HAV prevalence was observed in Prata village (96.0%) and Tocantinópolis downtown (96.1%) and the lowest prevalences were found in Mariazinha Village (74.2%) and Bela Vista Urban area (66.6%). Spatial distribution showed high prevalence of total anti-HAV in homes that received up to 500 reais (about 100 dollars) (Fig. 2).

IgM anti-HAV was detected in 15 individuals giving a prevalence of 1.7%. Among 15 acute cases, most of them were males (10/15), aged more than 41 years old and (10/15) lived at Folha Grossa Village (13/15). Real time PCR was done in positive IgM anti-HAV samples and all of them tested negative for HAV RNA.

### Discussion

The present study gives new information about HAV prevalence in indigenous population before universal HAV immunization in Brazil. Historical accounts indicate that all Apinajé indigenous have contact with Luso-Brazilian since the seventeenth century. In the present study, total anti-HAV prevalence among indigenous was 88.2%, almost the same found in urban population 85%, suggesting a pattern of homogeneous dispersion between both groups. Among Brazilian indigenous population, few studies reported HAV infection where prevalences varies from 93.7 to 100% [1, 11–13]. An earlier study in Brazil observed that indigenous aging less than 50 years from Parakana Bom Jardim village, who have never been in contact with Portuguese descent, showed no seropositive for HAV, while all indigenous (Novo Parakanã and Asurini Trocará) that had contact with Luso-Brazilian were soropositivity to HAV [12].

In the present study, there was a drop in total anti-HAV prevalence in children aging above 6 years and 100% of individuals aging more than 40 years were anti-HAV positive. Children aging 0 to 5 years had 41.1% of HAV prevalence what is lower than found among studies conducted in 2011 at children 1 to 4 years of age (16.67%) and in 2010 at children aging 1 to 5 years (22.4%) both located in North Region of Brazil [14, 15].

Total anti-HAV prevalences above 90% was found in indigenous tribes and non- indigenous settings demonstrating that infection is dispersed in this region. Lin et al. [16] demonstrated an overall prevalence of 98.1% of anti-HAV in indigenous from Taiwan and this rate did not differ between villages or gender, showing wide HAV dispersion between these locations. High prevalence of HAV was also observed in other indigenous population before universal immunization. Tsou et al. [17] found annual incidence of 2.96/100,000 inhabitants and this value dropped to 0.3/100,000 between 1995 to 2008 in Taiwan. This is the result of three dose schedule vaccination in indigenous children aging between 15 months to 12 years old in 1995 and two dose regimens applied in nine cities close to indigenous population in 1998. In Australia, after the death of indigenous children with hepatitis A in Queensland, it was adopted vaccination in the region, which drastically reduced hepatitis A incidence from 237 to 9 cases between 1996 and 2003 [18].

Total anti-HAV prevalence was elevated in indigenous and non-indigenous population that have low monthly income. This demonstrates that HAV is common in low resource areas independent of ethnic group. Some studies demonstrated that HAV prevalence is associated to water access and monthly income [19–21]. HAV dispersion could increase the probability of outbreaks as reported in several communities [5, 22–24] and favor person-to-person transmission of HAV [25] that could be one of the main route of HAV dissemination among person that have strict contact, as happening in the indigenous culture.

Although it was found high total anti-HAV prevalence, the prevalence of IgM anti-HAV was low (1.7%) and none of them had HAV RNA. Low prevalences of IgM anti-HAV were also found in Afro Brazilian isolated community (0.6%) [26], risk group in Nigeria (1.5%) [27] and plasma donors in China (0.7%) [28]. However, data from South Africa's National Health Laboratory Service found 3.3% of IgM anti-HAV between 2005 and 2015 [29] probably due to high risk observed in acute hepatitis cases. In indigenous community from North Brazil, more than 30% of acute HAV cases were found characterizing an outbreak [1] and a fatal course of hepatitis A was described in three indigenous children from western and northern Queensland, Australia, between 1993 and 1998 [30]. This situation demonstrates the importance of monitoring acute hepatitis cases in indigenous community.

Acute cases were high prevalent among males and those aging more than 40 years, where the disease is more severe. A study of hospitalization due to HAV in Taiwan found overall mortality rate of 16.8 per 1000 hospitalizations where high fatality rates was found in males and adjusted odds ratio for death rose by age and increased rapidly over 40 years of age [31]. Some studies showed that fatal cases occurred in individuals aging more than 40 years [32, 33]. High prevalence of acute cases was found in Folha Grossa village, indigenous tribe, that was not included as low monthly income, but it is near to urban area. A probable hypothesis is that one acute case had disseminated the virus in this community.

### Conclusion

It was observed high overall anti-HAV prevalence in this study, but it did not differ between Indigenous and non-indigenous communities and most of acute cases was found in individuals aging more than 40 years reinforce the importance of universal HAV immunization to reduce the transmission of the virus in this group.

## Limitations

The major limitation of the study is the lack of clinical data and risk factors of the population studied that could explain the high prevalence of hepatitis A in some communities.

## Data Availability

The datasets generated and/or analysed during the current study are not publicly available due to confidenciality and privacity requested by Ethics Committee and Institution that allowed the recruitment of volunteers but are available from the corresponding author on reasonable request.
